# Prevalence and Species Distribution of Neonatal Candidiasis: A Systematic Review and Meta-Analysis

**DOI:** 10.3390/diseases12070154

**Published:** 2024-07-12

**Authors:** Amr Molla, Muayad Albadrani

**Affiliations:** 1 Department of Medicine, College of Medicine, Taibah University, Madinah 42353, Saudi Arabia; 2 Department of Family and Community Medicine and Medical Education, College of Medicine, Taibah University, Madinah 42353, Saudi Arabia

**Keywords:** candida, candidiasis, prevalence, meta-analysis, Saudi Arabia

## Abstract

Background and aim: Candida infection is a significant cause of morbidity and mortality in neonatal intensive care units (NICU) globally. We aimed to conduct a systematic review to investigate the prevalence of candida among causative organisms of neonatal sepsis and identify the distribution of candida species infecting Saudi neonates. Methods: We comprehensively searched Web of Science, Scopus, PubMed, and Cochrane Library from their inception till November 2023. After screening titles, abstracts, and full texts, we ultimately included 21 eligible studies. The designs of the included studies were randomized clinical trials, cohorts, case–control, and case reports; the methodological quality was appraised using the Cochrane risk of bias assessment tool, NIH tool for observational studies, and Murad tool for assessing case reports. Results: Our systematic review and meta-analysis pooled data reported in 21 studies in the Saudi populations, which provided data on different types of candidal infections in 2346 neonates. The pooled data of ten retrospective studies enrolling 1823 neonates revealed that candida species resembled 4.2% of the causative organisms of neonatal sepsis among Saudi neonates (95%CI [2.5%; 5.9%], *p* = 0.000). Additionally, out of a total of 402 candida species that were identified among the included studies, *C. albicans* prevailed mostly among Saudi neonates, followed by *C. parapsilosis*, NS candida, and *C. tropicalis* (50.25%, 21.40%, 12.44%, and 9.45%, respectively). Conclusions: We found that candida species prevailed in 4.2% of 1823 cases of neonatal sepsis; the most common candida species was *C. albicans*. We could not pool data regarding risk factors or susceptibility of candida species to different treatment modalities due to insufficient data, requiring future large-scale, high-quality studies to be conducted.

## 1. Introduction

Candida infection is a significant cause of morbidity and mortality in neonatal intensive care units (NICU) globally [1,2]. Vulnerable premature and low birth weight infants are at exceptionally high risk, with reported candidiasis incidence rates ranging from 7 to 20% in developing regions [3,4,5]. Candida commonly colonizes the skin, mucosal surfaces like the oral cavities, gastrointestinal tracts, and vagina [1,6]. However, certain situations like reduced immune response, microflora disruption, and exposure to medical devices allow Candida overgrowth and potential dissemination [7,8]. Invasive candidiasis is defined as candida infections involving normally sterile blood, tissues, bones, and organs [1], while non-invasive, superficial Candida infections may present as oral thrush or diaper dermatitis [9,10]. Beyond mortality threats, both invasive and superficial neonatal candidiasis correlate to worsened neurodevelopment among survivors [11].

Globally, candida remains the third most prevalent cause of neonatal late-onset sepsis, behind Staphylococcus and Klebsiella bacteria [11,12]. In developing regions, reported incidence rates of combined invasive and superficial candidiasis infections in neonatal intensive care units range widely from around 3% to over 25% [2,4,13]. Premature rupture of membranes, very low birth weight, use of broad-spectrum antibiotics and steroids, fungal colonization, surgery, endotracheal intubation, underlying gastrointestinal disorders, and central vascular access heighten vulnerability [14]. Despite antifungal treatment, attributable mortality remains high between 10 and 40% [15]. Beyond death, neurodevelopment impairment among survivors presents an equally alarming longer-term burden [16].

Saudi Arabia is a critical focal area for tackling neonatal candidiasis, given the estimated higher national incidence rates than in Western Europe and North America [17]. Local single-center reports suggest between 5 and 20% of neonatal sepsis cases show confirmed Candida infections depending on geographical area [18,19]. However, substantial gaps exist regarding the large-scale epidemiological synthesis of neonatal candidiasis prevalence across Saudi Arabia using consolidated national data.

Therefore, this systematic review and meta-analysis aim to thoroughly investigate the existing literature on neonatal candidiasis in Saudi Arabia. We aim to assess the overall prevalence of candida among other causative organisms of neonatal sepsis, identify the common candida species infecting neonates, and identify primary risk factors, standard treatment regimens, and attributable mortality rate on a national level.

## 2. Method

Our systematic review and meta-analysis (SRMA) was crafted following the most recent Cochrane Handbook guidelines [20] and reported as per the Preferred Reporting Items for Systematic Reviews and Meta-Analyses (PRISMA) checklist [21].

## 3. Literature Search

We conducted a comprehensive search of databases including Scopus, Web Of Science, PubMed, Google Scholar, and the Cochrane Library from their inception until November 2023 using the following keywords: ((((Neonates OR Neonate OR neonatal OR infant OR fetal OR newborn OR Premature OR Preterm OR Fetal OR postmature OR dysmature OR dysmaturity OR (small for gestational age) OR (low birth weight) OR LBW OR (intrauterine growth restriction) OR IUGR)) AND (candida OR candidiasis OR candida OR candidemia OR monilia OR monilial OR moniliasis OR thrush)) AND (Saudi OR “Saudi Arabia” OR “Kingdom of Saudi Arabia” OR KSA OR SA OR Riyadh OR Medina OR Mekka)). The search results were compiled using EndNote software (Version 20.2.1), duplicates were removed, and the remaining studies were transferred to an Excel sheet for further screening.

## 4. Inclusion and Exclusion Criteria

We included any English-language study in our SRMA that met the following criteria: population—neonates from Saudi Arabia aged less than one month; exposure—any superficial or deep candidal infection; control—not specified; outcome—any safety and efficacy outcomes; study designs—randomized clinical trials (RCTs), cohorts, case–control, case series, or case reports. Studies were excluded if they defined their neonatal population with an age range that exceeded one month, combined neonates and infants into one group, studied a mixed population of Saudi and non-Saudi subjects, were not written in English, or were animal studies.

## 5. Data Extraction

We collected data about study ID (last name of first author–publication year), title, study design, initiation date, termination date, study duration, hospital name, Saudi city, primary neonatal pathology/outcome and its definition by the authors, events of candidal infection, total study population, sex, candida species, birth weight, mode of delivery, and conclusion.

## 6. Assessment of Risk of Bias

Our SRMA incorporated RCTs, cohorts, cross-sectional studies, and case reports. The RCTs were evaluated methodologically using the first version of the Cochrane Risk of Bias (ROB) tool [22]. This tool comprises seven domains: selection, performance, detection, attrition, and reporting biases. Each domain was assessed as having a high, low, or unclear risk of bias. The methodological quality of the cohorts and cross-sectional studies was assessed using the NIH tool [23], which consists of 14 questions, each scored as zero, 0.5, or one. The overall study quality was classified as poor, fair, or good based on the total score (0–7 points, 7.5–10.5 points, or 11–14 points, respectively). Case reports were evaluated using the Murad et al. quality appraisal tool [24], which includes four main domains of selection, ascertainment, causality, and reporting, investigated through eight questions. Each study was assigned a score of eight, denoting it as poor, fair, or good quality.

## 7. Data Synthesis

We analyzed the available categorical outcomes as pooled proportions with 95% CIs; the random effect model was applied. We investigated the statistical heterogeneity between studies using the I^2^ statistics Chi-squared test, with *p* < 0.1 considered heterogeneous and I^2^ ≥ 50% suggestive of high heterogeneity. We conducted statistical analyses using Comprehensive Meta-Analysis Software (Version 3) (CMA, Englewood, NJ, USA).

## 8. Result

### 8.1. Literature Review

**Figure 1:**
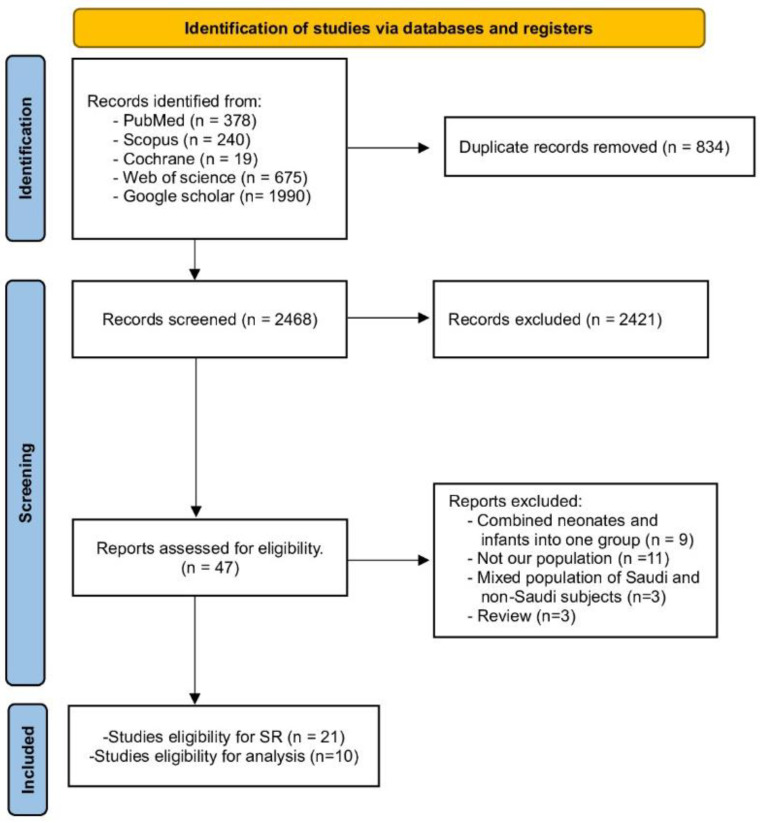
PRISMA flow chart.

A thorough search was conducted across various databases, including WOS, PubMed, Scopus, Cochrane Library, and Google Scholar. This search yielded 3302 records in total. After removing duplicates, 2468 studies remained. These were screened based on their title and abstracts. Of these, 47 studies were further scrutinized by examining their full texts for relevance. Ultimately, 21 studies were selected for our Systematic Review. Out of them, only ten studies were included in the analysis. Further details can be found in Figure 1 (PRISMA).

### 8.2. Characteristics of the Studies

**Figure 2:**
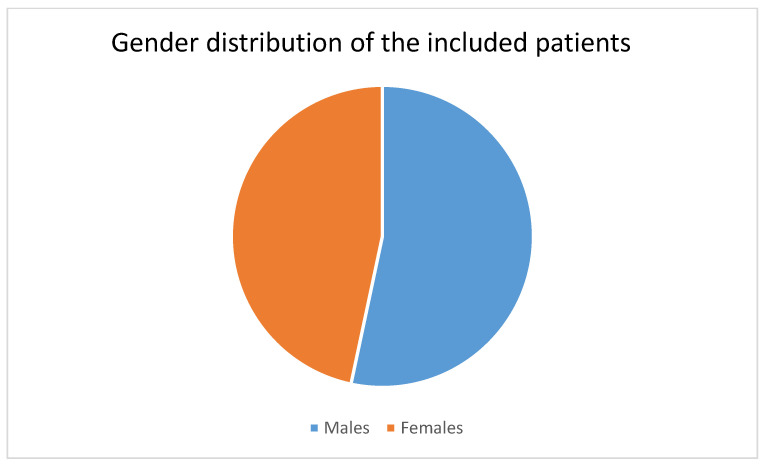
Distribution of males and females in the included patients.

This SRMA incorporated data from 21 studies [18,25,26,27,28,29,30,31,32,33,34,35,36,37,38,39,40,41,42,43,44] conducted in Saudi Arabia. These studies, which spanned various cities, including Aseer, Jeddah, Medina, Qatif, Tabuk, and Taif, provided data on different types of candidal infections in 2346 neonates. The majority of these studies, 14 in total, were carried out in the city of Riyadh. The studies had an average duration of 3.8 years, ranging from 1 year to 7.25 years. Approximately 50% of the population studied were male neonates (Figure 2). Additional details regarding the characteristics of the eligible studies and populations are presented in Table 1.

### 8.3. Assessment of Studies Quality

This SRMA included two Randomized Controlled Trials (RCTs) [26,29], 15 cohort studies [18,25,27,28,30,31,32,35,36,37,38,39,40,41,42], one case–control study [43], and three case reports [33,34,44]. The Murad tool assessed the case reports, which indicated either poor [34,44] or fair methodological quality [33]. The RCTs were evaluated to have an unclear risk of bias. The 15 cohort studies and one case-control study were judged to be fair or good quality. Supplementary Tables S1–S3 provide more details.

### 8.4. Prevalence of Candida among Causative Organisms of Neonatal Sepsis

**Figure 3:**
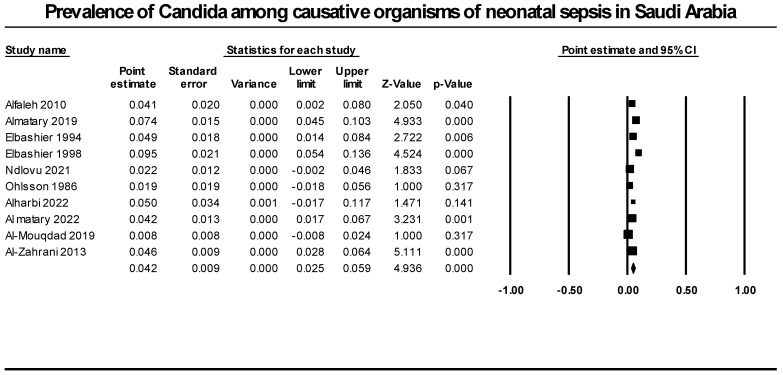
Prevalence of candida among causative organisms of neonatal sepsis in Saudi Arabia. Included studies (Alfaleh 2010 [37], Almatary 2019 [19], Elbashier 1994 [30], Elbashier 1998 [31], Ndlovu 2021 [28], Ohlsson 1986 [29], Alharbi 2022 [18], Al matary 2022 [40], Al-Mouqdad 2019 [39], Al-Zahrani 2013 [37]).

We synthesized data from ten retrospective studies that included 1823 neonatal infection cases to determine the prevalence of candida as a cause of neonatal sepsis. Our analysis revealed that candida species prevalence accounted for 4.2% of all neonatal sepsis causative agents (95% CI [2.5%; 5.9%], *p* = 0.000). We detected significant heterogeneity that could not be resolved (*p* = 0.0001, I^2^ = 72.1%) (Figure 3).

### 8.5. Distribution of Candida Species among Saudi Neonates in Different Cities

**Figure 4:**
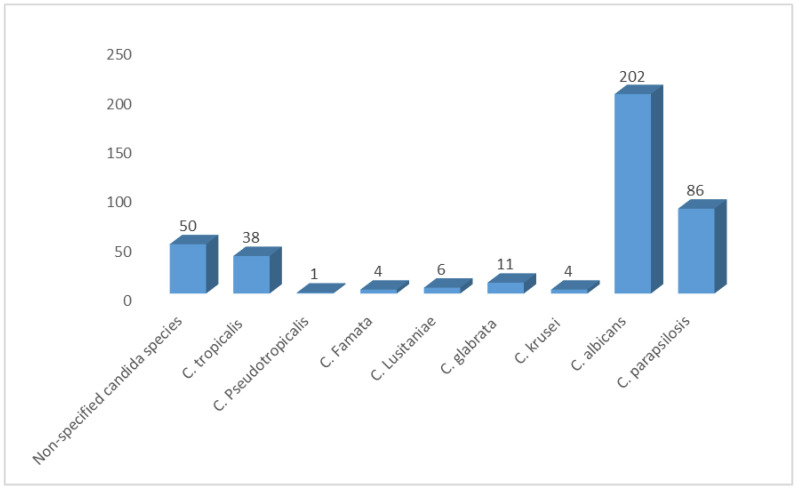
Distribution of Candida species among Saudi neonates in Saudi Arabia.

We pooled data from 21 studies and identified 402 yeasts isolated from neonates. Our SRMA showed that *C. albicans* was the most prevalent Candida species, representing 50.25% of all isolates. *C. parapsilosis* and *C. tropicalis* followed, representing 21.40% and 9.45%, respectively. Non-specified candida species (NS candida) represented 12.44% of the identified isolates. Moreover, we found a low prevalence of some species, such as *C. glabrata, C. lusitaniae, C. krusei, C. famata, and C. pseudotropicalis* (2.73%, 1.5%, 0.99%, 0.99%, 0.25%, respectively) (Figure 4).

**Figure 5:**
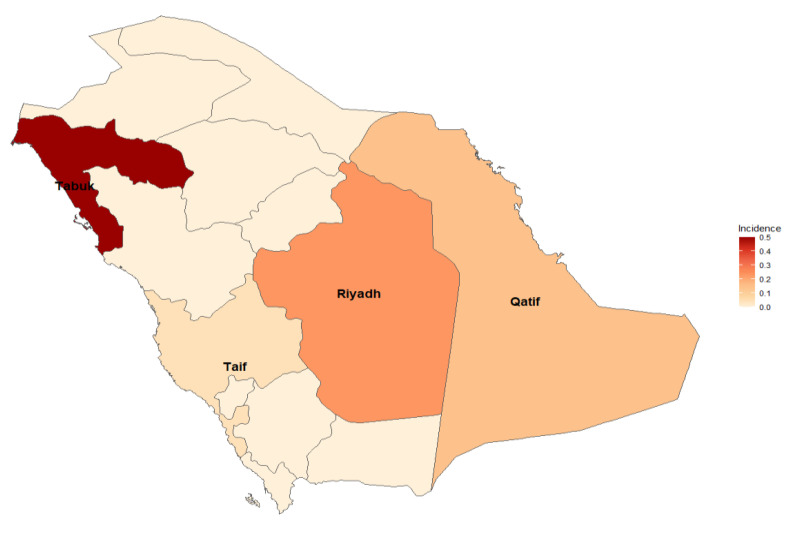
Different regions in Saudi Arabia with higher incidences of Candida infections.

Nevertheless, neonatal Candida infections were observed in various cities. Notable findings include Qatif, where approximately 7.58% of neonates were affected, and Riyadh, with an incidence of approximately 4.66% of neonates. Tabuk reported a lower incidence of approximately 2.17, while Taif had a slightly higher incidence of approximately 4.55%. These findings emphasize the importance of monitoring and managing Candida infections in neonates across different regions (Figure 5).

### 8.6. Invasive Candidiasis

Four studies reported data on 245 Saudi neonates from Aseer, Medina, and Riyadh with invasive candidiasis. Al-Jasser and colleagues investigated the distribution of candida species across different age groups and found 17 cases of invasive candidiasis in NICU over six years. *C. albicans* (13 cases) was the predominant candida species in the study population [41]. Another study conducted in Aseer Central Hospital compared the safety and efficacy of amphotericin B and caspofungin for treating 32 cases of neonatal invasive candidiasis, of which *C. albicans* was isolated from 24 cases. The results indicated that caspofungin was superior, achieving a favorable response in 86.7% of patients, compared to only 41.7% in the amphotericin B group (*p* = 0.04). Moreover, the caspofungin group had a significantly lower incidence of adverse events than the amphotericin B group [26]. Almoosa et al. identified 66 cases of invasive candidiasis among King Fahad Medical City neonates over five years. The most common candida species were *C. albicans*, *C. tropicalis*, *C. parapsiolosis*, *C. lusitaniae*, and *C. famata* (29 cases, 11 cases, 11 cases, four cases, three cases, respectively) [35]. Eisi et al. conducted a retrospective study and found 130 cases of neonatal candidiasis in the Madinah Maternity and Children Hospital NICU over seven years. The study classified neonates based on deep tissue involvement. Deep tissue invasion was found in 16 cases. It was significantly associated with a higher risk of cerebral palsy, heart failure, and more extended hospital stay than the non-deep tissue invasion group [32].

### 8.7. Other Forms of Candidal Infection/Involvement in the Saudi Neonates

The prevalence and impact of candidal infection in Saudi neonates is a topic of interest for several studies. Mersal et al. compared nystatin and fluconazole as prophylactic agents for candidal colonization in preterm or low birth weight infants. They randomly assigned 57 neonates to either group and found no difference in safety outcomes or invasive candidal infection rates. Nystatin was also more cost-effective than fluconazole [29].

Al-Hussaini et al. examined the candida species and their antifungal susceptibility in 100 neonates. They reported a colonization rate of 51%, with higher rates in preterm neonates. The most common sites of colonization were the perianal area and oral cavity; the most common species was *C. albicans* (58.8%). The isolated candida species showed moderate sensitivity to fluconazole, itraconazole, and ketoconazole (68.6%, 80%, and 64.7%, respectively) while showing low sensitivity to amphotericin B (33%) [36].

Other studies explored the role of candida in neonatal conjunctivitis and ventilator-associated pneumonia (VAP). Faraz found that candida accounted for 3.37% of neonatal conjunctivitis cases [25], while Afify et al. found that candida accounted for 9% of neonatal VAP cases. They suggested that candida infection was a significant risk factor for VAP in neonates [42].

Some case reports illustrated the challenges of diagnosing and treating different candidal infections in Saudi neonates. Abuhajj and his team highlighted a case of congenital cutaneous candidiasis, which can often be mistaken for benign or bacterial eruptions. They described a neonate with a congenital maculopapular eruption that spanned the torso, diaper area, extremities, palms, and soles. The neonate exhibited leukocytosis (>30 k) and bilateral lung opacifications. Initial treatment with empirical antibiotics exacerbated the condition. However, a significant improvement was observed just three days after initiating intravenous amphotericin B and topical miconazole, pending the results of a skin-scraping culture. After two weeks of treatment, the neonate’s skin returned to normal, and the child was discharged in stable condition [43]. Azhar et al. reported a case involving a female neonate who was admitted to the hospital with symptoms including fever (38.1 °C), leukocytosis, tachycardia (168 bpm), and hypotension. Initially treated as a septic shock with IV fluids, empirical ampicillin, and cefotaxime, subsequent chest X-rays revealed a sizeable cardiac shadow. This was attributed to vegetation in both the left and right ventricles and a thickened pericardium, as detected by echocardiography. Following the administration of intravenous fluconazole and amphotericin B, the size of the vegetation decreased until it completely disappeared, as confirmed by an echocardiography after 40 days. The patient was discharged following clinical improvements [33].

Al-Arishi documented a case of a male neonate with a severely dysplastic right kidney and a moderately hydronephrotic left kidney. A left cutaneous pyelostomy was performed on the fourth day of life due to declining renal function. Urine analysis from the pyelostomy catheter revealed numerous yeast cells, and a complete blood count and blood samples confirmed the presence of candidemia. Treatment with Ambisome rendered the blood completely sterile after just five days of initiation [34].

## 9. Discussion

Our study included 21 studies, encompassing a total of 2346 neonates. The consolidated data from ten retrospective studies, which enrolled 1823 neonates, indicated that candida species accounted for 4.2% of the causative organisms of neonatal sepsis among Saudi neonates. Out of the 402 candida species identified in the included studies, *C. albicans* was the most prevalent among Saudi neonates, followed by *C. parapsilosis*, NS candida, and *C. tropicalis*. Furthermore, a literature review revealed that four studies reported 245 neonates diagnosed with invasive candidiasis, with candida accounting for 3.37% and 9% of organisms causing neonatal conjunctivitis and Ventilator-Associated Pneumonia (VAP), respectively.

A previous multicenter study conducted in eight Arab countries over 19 years (1990–2009) enrolled data on sepsis from 2308 neonates [44]. However, they found inadequate data regarding the prevalence of candida among other microbiological causes of sepsis. They concluded that Candida species had been emerging in multiple Arab countries, including Egypt, Bahrain, UAE, and Kuwait; a study conducted in Kuwait stated that candida represented 14% of the causes of neonatal sepsis [45]. Additionally, a study by Pillay and colleagues, conducted over three years and enrolling 681 cases of neonatal sepsis, reported results consistent with ours, with fungal isolates accounting for 4.5% of cases of neonatal sepsis and the majority of cases being bacterial (over 95%) [46].

We found that *C. albicans*, *C. parapsilosis*, and *C. tropicalis* were the most common yeast isolated from 402 neonates (50.25%, 21.40%, and 9.45%, respectively). Moreover, we found a low prevalence of some species such as *C. glabrata, C. lusitaniae, C. krusei, C. famata, and C. pseudotropicalis* (2.73%, 1.5%, 0.99%, 0.99%, 0.25%, respectively). Non-specified candida species (NS candida) represented 12.44% of the identified isolates. This was consistent with what was reported by an 11-year, multinational, retrospective study with participants from 23 different European countries. They found that among 422 neonates with candidal infections, *C. albicans*, *C. parapsilosis*, and *C. tropicalis* were the most common species, prevailing in 60.1%, 27.7%, and 2.8%, respectively [47]. Another study by Cook enrolled 127 neonates from eight countries and found that the most common isolated candida species were *C. albicans*, *C.parapsilosis*, and *C. auris* (35%, 30%, and 14%, respectively) [48].

However, a three-year study conducted in South Africa found that *C. parapsilosis* was the most prevalent, followed by *C. albicans* and NS candida (45.2%, 29%, and 25.8%, respectively) [46]. These discrepancies could be attributed to the different demographics, narrower time scale of the study, and a small sample of 31 candida species. Our study pooled data over twenty years and enrolled over 400 candida species among neonates.

We could not pool and analyze data regarding possible risk factors for neonatal candidiasis or susceptibility of candida species to different treatment modalities. However, some included studies provided data regarding significant risk factors for different candidal infections. Eisi and colleagues found that gestational age of less than 32 weeks and candidal infection of central venous catheters were significantly associated with deep candidal infections [32]. Additionally, Afify et al. found that the nosocomial infection by candida was a risk factor for developing ventilator-associated pneumonia (VAP), in addition to other factors, including prolonged NICU admission, invasive maneuvers, hypothermia, high CRP, and hypoalbuminemia [42]. The literature review provided additional insights about other possible risk factors for candidal infection in neonates. A multicenter study by Xia and colleagues found that over 90% of neonates diagnosed with invasive candidiasis were administered an extensive course of broad-spectrum antibiotics.

Moreover, about 70% and 60% of the neonates were either low birth weight or had central venous catheters, respectively [49]. Manzoni et al. confirmed that a central venous catheter posed a significant risk of developing candidal infection [50]. The influence of low birth weight, central venous catheter, and prior extensive antibiotic course and their significant relation to candidal infection in neonates was confirmed by Benjamin Jr. [51].

Regarding treatment modalities for neonatal candidiasis, Mersal et al. compared nystatin and fluconazole as prophylactic agents for candidal colonization in preterm or low birth weight infants. They randomly allocated 57 neonates to either group and found no difference in safety outcomes or rates of invasive candidal infection. Nystatin also proved to be more cost-effective than fluconazole [29].

A study by HSU analyzed 342 episodes of candidiasis in neonates and children and reported that 97.1% received antifungal treatment, but 2.9% were untreated due to patient demise before proceeding to treatment. Antifungal therapy commenced, on average, 1.81 days after obtaining diagnostic cultures, notably delayed in neonates compared to children (2.1 vs. 1.7 days). The mean duration of antifungal therapy was 18.5 days. Among the 332 treated episodes, 45.5% experienced regimen modifications, primarily due to poor initial response (66.9%), suspected resistance (23.8%), or undocumented reasons (9.3%). Initial prescriptions favored fluconazole (62.3%), followed by amphotericin B (24.7%) and caspofungin (4.5%), while final regimens were fluconazole/voriconazole (39.5%), amphotericin B (29.2%) and echinocandin (28.9%). Catheter removal within three days occurred in only 32.2% of cases. Neonates faced prolonged fungemia, higher treatment failure rates (31.0% vs. 19.7%), and increased sepsis-related mortality (28.3% vs. 17.5%) and in-hospital mortality (42.7% vs. 25.4%) post-invasive candidiasis compared to children. Susceptibility studies on 295 isolates revealed a 14.6% fluconazole-resistant or susceptible-dose-dependent candida rate, with no significant differences between neonates and non-neonatal pediatric cases [52].

We conducted the first systematic review and meta-analysis (SRMA) to evaluate the incidence of candidal infections in neonates from Saudi Arabia. We amalgamated data from 2346 neonates across 21 studies. These studies were carried out in diverse Saudi cities such as Riyadh, Jeddah, Medina, Qatif, Tabuk, and Taif, bolsters our findings’ applicability to the entire Saudi population. We confined our data inclusion to neonates, which were either explicitly defined as less than one month old or as neonates without an age specification. We excluded any study that defined neonates as older than one month or included a mixed-age group. Nonetheless, our study had certain limitations. The data we pooled spanned a considerable period from 1989 to 2020, which could lead to inconsistencies concerning the diagnostic laboratory methods and identification of various candida species. Most of the included studies were conducted in Riyadh City, which might limit the applicability of our findings to the entire Saudi population. Our data included three case reports and several studies over two decades. Despite these limitations, we endeavored to compile all accessible evidence to provide a comprehensive overview of the prevalence of candidal infections among Saudi neonates. However, we could not pool and analyze data about potential risk factors for neonatal candidiasis or the susceptibility of different candida species to various treatment modalities. We recommend conducting comprehensive future studies to enhance our understanding of Candida species prevalence among neonates in Saudi Arabia. Additionally, exploring antifungal resistance in greater detail for the identified Candida species would be valuable.

## 10. Conclusions

In conclusion, our study evaluated the epidemiology of Candida species among neonates in Saudi Arabia. We found that candida species were present in 4.2% of 1823 neonatal sepsis cases, based on data from ten retrospective studies conducted between 1980 and 2020 in Aseer, Jeddah, Medina, Riyadh, Qatif, Tabuk, and Taif. Among the 402 candida isolates identified, *C. albicans* was the most common, followed by *C. parapsilosis* and *C. tropicalis*. Unfortunately, due to insufficient data, we could not pool information regarding risk factors or the susceptibility of different candida species to various treatment modalities. We advocate for future large-scale, high-quality studies to provide detailed insights for pediatricians and primary care physicians regarding neonatal candidal infections in Saudi neonates.

## Figures and Tables

**Table 1:** Summary and baseline characteristics of the included studies.

Study ID	Study Design	City of Saudi Arabia	Hospital Name	Total Population	Males, %	Birth Weight (g) Mean +/− SD	Mode of Delivery	Initiation Date/ Termination Date	Study Duration	The Main Neonatal Pathology/Outcome of the Study	Diagnostic Methods	Number of Candida Isolates	Candida Species	Conclusions
Mohamed 2012 [26]	RCT	Aseer	Aseer Central Hospital	32	56.25%	877.646 ± 80.81	-	October 2008/ September 2010	Two years	Treatment of invasive candidiasis	Neonates are confirmed with invasive candidiasis if they have at least one positive blood culture and/or positive cerebrospinal fluid culture or positive urine culture obtained by suprapubic aspiration.	32	1. *C. albicans* = 24 2. *C. parapsiolosis* = 5 3. *C. tropicalis* = 3	“Caspofungin is more effective, safer, and alternative to amphotericin B for treating invasive candidiasis in newborn infants”.
Azhar 2012 [33]	Case report	Jeddah	King Abdulaziz University Hospital Pediatric Emergency Facility	1	0	1400	Emergency cesarean section	-	-	Fungal pericarditis and endocarditis	-	1	*C. albicans*	“Fungal endocarditis in neonates is a rare, serious disease that has a poor prognosis if not treated properly. Interestingly, this disease is increasingly frequent in today’s highly advanced medical practices globally. Recognizing FE early is challenging due to its nonspecific symptoms, but an accurate diagnosis can be made with a high index of suspicion and understanding of the predisposing factors. Management with antifungal medications can be an adequate choice, even in critically ill patients”.
Eisi 2022 [32]	Retrospective study	Medina	Madinah Maternity and Children’s Hospital	130	52.30%	1350 ± 113	Vaginal delivery: 61.5%	January 2012/ December 2019	Seven years	Invasive candidiasis	Deep tissue Candida invasion was diagnosed based on radiological or histopathological evidence of Candida seeding in the central nervous system, eyes, heart, lungs, liver, spleen, kidneys, joints, or long bones. Two cases of deep tissue Candida invasion were identified based on reviewing the notes and the diagnosis made by the treating physicians for each infant.	130	1. *C. albicans* = 59 2. *C. parapsilosis* = 55 3. *C. krusei* = 3 4. *C. tropicalis* = 12 5. *C. glabrata* = 1	“Persistent Candida growth in blood cultures, prematurity, and long-term antibiotic use are significant risk factors for deep tissue Candida invasion. Deep tissue Candida invasion is associated with prolonged hospital stay and higher neonatal morbidity”.
Elbashier 1994 [30]	Retrospective study	Qatif	Qatif Central Hospital	144	64.60%	-	-	January 1989/ January 1992	Three years	Neonates with septicemia	The diagnosis was made based on clinical and laboratory parameters. Blood culture isolates were classified either as clinically significant isolates or as contaminants. A positive culture was considered clinically significant only when at least one positive blood culture (and/or CSF) was obtained with supporting clinical findings or laboratory data. On the other hand, a contaminant was an isolate obtained in their absence, i.e., where the clinical and laboratory findings suggested that the organism was not involved in any infection. Early-onset infection was defined as infection occurring within the first 48 h of life, and late-onset infection was defined as infection occurring after that period.	7	1. *C. albicans* = 7	“We conclude that the incidence of neonatal septicemia, the prevalence of particular organisms, case mortality rate, and antimicrobial susceptibility vary greatly between hospitals in the same area, between different regions in the same country, and between countries. Extrapolation of data from one hospital to another is likely misleading: treatment should, if possible, always be based on local data”.
Elbashier 1998 [31]	Retrospective study	Qatif	Qatif Central Hospital (QCH) is the same hospital with intersecting duration with the above study	199	-	-	-	May 1990/ May 1995	Five years	Bloodstream infection in neonates	An episode of bacteremia was defined by the isolation of one (antimicrobial) or more (polymicrobial) microorganisms from blood culture(s), together with clinical evidence of systemic infection. Bacteremia, which developed before or within three days of admission and was not related to medical procedures undertaken after admission, was designated as community-acquired, and that with late-onset or indirect relation to medical procedures undertaken in the hospital was considered to be hospital-acquired.	19	NS candida	“Bloodstream infections continue to be life-threatening. Their incidence, the prevalence of causative organisms, and the case mortality rate vary greatly between hospitals in the same area, regions in the same country, and different countries. Extrapolation of data from one hospital to another may sometimes be misleading. Treatment should, as far as possible, be based on local data, and since that may also vary from time to time, even in the same hospital, regular surveillance of BSI is desirable”.
Alfaleh 2010 [37]	Retrospective study	Riyadh	King Khalid University Hospital.	98	48%	980	Vaginal 63%	January 2006/ December 2008	Three years	Late-onset neonatal sepsis (LOS)	Late-onset sepsis was defined as a positive result on one or more blood or cerebrospinal fluid (CSF) cultures obtained after 48 h of life. We took no measures to differentiate definite from possible infections with coagulase-negative Staphylococci (CONS) (i.e., repeated cultures from different sites or at a later date)	4	1. *C. albicans* = 2 2. *C. parapsilosis* = 2	“The rate of LONS was high and exceeded internationally reported rates in our tertiary care NICU. Gram-positive organisms continue to be major causative isolates. High priority should be placed on preventative steps to control nosocomial sepsis”.
Al-Matary 2019 [40]	Retrospective study	Riyadh	King Fahad Medical City	298	56.40%	1. Thirty-three neonates = 2187.3 ± 964.1 g for a neonate with EOS 2. A total of 265 neonates = 1501.1 ± 876 g for a neonate with LOS.	-	January 2011/ December 2015	Five years	Early- and late-onset neonatal sepsis	Early-onset sepsis (EOS) occurs in the first 72 h of life, or late-onset sepsis (LOS) occurs after 72 h of life. EOS is mainly due to organisms acquired before and during delivery, while LOS is due to organisms acquired after delivery and is mainly referred to as a healthcare-associated infection (HAI)	22	1. *C. albicans* = 14 2. *C. parapsilosis* = 4 3. *C. tropicalis* = 2 4. *C. glabrata* = 2	“Concerted efforts are needed to determine the spectrum of risk factors and the clinical characteristics of EOS and LOS to implement appropriate treatment strategies as sepsis remains a danger to neonatal wellbeing. Moreover, our study emphasizes that using aminoglycosides is much more agreeable than the broad-spectrum antibiotics which are more widely used nowadays”.
Ohlsson 1986 [27]	Retrospective study	Riyadh	King Faisal Specialist Hospital	53	-	-	-	November 1980/ October 1984	Four years	Neonatal septicemia	In all cases of suspected sepsis, cerebrospinal fluid, stools, and urine cultures were obtained by suprapubic aspiration.	1	1. *C. pseudotropicalis* = 1	“The incidence of neonatal sepsis among patients born in the hospital was 2.5/1000 live births. Mortality from sepsis was 33% and was associated with neutropenia in 63%. The most commonly isolated bacteria were *E. coli*, *Klebsiella*, and *Staphylococcus aureus*. *Salmonella enteritidis* serotypes were isolated in 4% of the cases. Group B streptococci (GBS) were isolated, for the first time, from the blood of 3 neonates. *Salmonella* species were less frequent, and GBS was more often isolated than previously. GBS have now appeared as an etiologic organism in neonatal sepsis, as well as in Saudi Arabia. *Salmonella septicemia* remains more common in Saudi Arabia than in the West”.
Alharbi 2022 [18]	Retrospective study	Riyadh	King Abdulaziz University Hospital	40	47.60%	-	-	May 2011/ October 2018	7.25 years	Neonatal sepsis	all neonates (0–28 days of age) born in or admitted to the hospital with clinically diagnosed sepsis and who had a blood culture test were included in this study. Sepsis was clinically diagnosed if the infant presented with signs and symptoms of systemic inflammatory response syndrome (SIRS) attributed to microbial etiology, whether confirmed microbiologically or not.	2	NS candida	“This study points to a likely emergence of coagulase-negative *Staphylococci* as the main cause of sepsis among neonates. Ampicillin and gentamicin are highly effective against the commonly isolated bacterial pathogens that cause neonatal sepsis”.
Al-Matary 2022 [41]	Retrospective study	Riyadh	King Fahad Medical City	237	52.70%	58.6% had birth weights of <1.5 kg	61.6% had CS	January 2016/ May 2020	4.33 years	Neonatal sepsis	-	10	NS candida	“Early antibiotic administration in patients with neonatal sepsis can improve the survival rate and reduce the incidence of complications”.
Al-Mouqdad 2019 [39]	Retrospective study	Riyadh	tertiary hospital in Saudi Arabia? King Saud Medical City,	132	44.50%	917.95 ± 355.95	-	January 2014/ December 2017	Three years	Neonatal sepsis	Clinical (suspected) sepsis: The exact definition of suspected neonatal sepsis remains vague. As pro-inflammatory solid cytokines may influence the clinical features of sepsis, clinicians rely on clinical features in their decision to suspect sepsis and start antimicrobial agents. Confirmed (proven) sepsis: Detection of a pathogen (positive culture) in otherwise sterile body fluid and clinical and laboratory signs of sepsis. Early-onset sepsis: Sepsis is caused by pathogens transmitted vertically from mother to infant in the first three days of life. Late-onset sepsis: sepsis caused by horizontally acquired pathogens that occur after three days of an infant’s life.	1	NS candida	“In our center, amikacin–cloxacillin combination therapy was associated with lower mortality in deficient birth weight neonates with late-onset sepsis compared with cefotaxime–ampicillin therapy”.
Faraz 2019 [25]	Prospective study	Riyadh	King Khaled Hospital Majma’ah	84	51%	-	-	January 2016/ January 2018	Two years	Conjunctivitis	-	3	NS candida	“Ophthalmia neonatorum is a common infection in newborns; gram-negative *Enterobacteriaceae* and gram-positive cocci are common etiological agents”.
Alhussaini 2016 [36]	Retrospective study	Riyadh	Saudi Arabian hospital	100	52%	1. <1.5 kg: 24% 2. >1.5 kg: 76%	Vaginal 16%	September 2014/ October 2015	One year	Early and late-onset candidal colonization	A positive surveillance culture defined fungal colonization at any time during their stay in NICU or at baseline. Thirteen early colonizations of *Enterobacteriaceaeion* were considered when Candida species were isolated from the initial cultures. In contrast, late colonization was considered when the initial cultures were negative and at least one subsequent culture was positive.	51	1. *C. albicans* (n = 30) 2. *C. tropicalis* (n = 9) 3. *C. glabrata* (n = 8) 4. *C. krusei* (n = 1) 5. *C. lusitaniae* (n = 2) 6. *C. parapsilosis* (n = 1)	“Candida has emerged as a common cause of infections in infants admitted to NICU, and *C. albicans* is the most commonly isolated candidal species. Neonatal infections caused by non-albicans species occur at a later age during their stay in NICU”.
Abuhajj 2021 [44]	Case report	Riyadh	Neonatal Intensive Care Unit, Arryan Hospital	1	1 (100%)	2540	Vaginal	-	-	Congenital cutaneous candidiasis	-	1	NS	“This report highlights that newborns could have CC without obvious risk factors such as premature rupture of membrane or positive maternal vaginal swab, and it could be misdiagnosed as benign skin eruption. Worsening of respiratory symptoms on top of the skin rashes is a potential presentation. Therefore, it is crucial to include CC screening as a part of unexplained skin rashes in neonates. Early diagnosis and treatment could reduce morbidity and prevent further complications”.
Al Arishi 1997 [34]	Case report	Riyadh		2	1 (100%)	2600	-	-	-	Candidemia afer pyelostomy	-	2	*C. parapsilosis*	“L-amp B may be a therapeutic option for therapy of invasive fungal infections in neonates who are at high risk for nephrotoxicity and other amp B-related adverse events. Studies are needed to determine the pharmacokinetics, safety, efficacy, and optimal therapeutic dosage of L-amp B in this patient population”.
Almoosa 2017 [35]	Retrospective study	Riyadh	King Fahad Medical City	66	-	-	-	January 2010/ January 2015	5 years	Invasive candidiasis	Invasive Candidiasis was defined as the isolation of Candida from blood, cerebrospinal fluid (CSF), or other sterile body fluids such as synovial fluid, peritoneal fluid, and pleural fluid.	66	1. *C. albicans* = 29 2. *C. tropicalis* = 11 3. *C. parapsilosis* = 11 4. *C. famata* = 3 5. *C. lusitaniae* = 4 6. Other = 8	“*Candida albicans* is the most common isolate among all Candida species. Gender, low birth weight, prolonged ICU stay, presence of vegetation, positive blood culture, and mechanical ventilation are strong predictive risk factors for death in children with invasive candidiasis. This finding could be applied as a prophylactic indicator in critically ill children, especially neonates”.
Al-Jasser 2004 [42]	Retrospective study	Riyadh	Armed Forces Hospital	17	-	-	-	1996/ 2002	Six years	Candidemia in NICU	Reported the laboratory method of identification only.	17	1. *C. albicans* = 13 2. *C. tropicalis* = 1 3. *C. parapsilosis* = 3	“These findings reinforce the need for continued and active surveillance programs to address the changes in the species distribution among candidal bloodstream isolates, which will help to develop effective, preventive, and therapeutic strategies”.
Mersal 2013 [29]	RCT	Riyadh and Jeddah	King Faisal Specialist Hospital and Research Cente; Maternity and Children Hospital.	57	45.60%	1. Twenty-four neonates = (1.16 ± 0.14) 2. Thirty-three neonates = (1.02 ± 0.2)	-	February 2011/ February 2012	One year	Neonatal antifungal prophylaxis	-	6	NS candida	“Intravenous fluconazole and oral nystatin at the prophylactic doses are equally effective and safe in preventing (ICI) in preterm neonates. However, oral nystatin is readily available, easily administered with a lower cost per neonate”.
Ndlovu 2021 [28]	Cross-sectional study	Tabuk	-	138	-	-	-	-	Two years	Nosocomial infections in NICU	-	3	1. *C. famata* = 1 2. *C. parapsilosis* = 2	“Most infections, 59.3%, occurred at less than 14 days of life. The most common site of infection was blood at 79.7%, and the most prevalent organisms were *Pseudomonas aeruginosa* and Methicillin-resistant *Staphylococcus aureus* at 22.5%, respectively. Some of the recommendations from the study are that healthcare workers should take extra precautions in collecting blood samples, ensure proper hand hygiene, and have external practitioners observe hand hygiene practices in NICU”.
Al-Zahrani 2013 [38]	Retrospective study	Taif	King Abdul Aziz Specialist Hospital	484	53.30%	1. G I (<1000) 29.54% 2. G II (1000–1500) 27.9% 3. G III (1501–2500) 28.5% 4. G IV (>2500) 14%	-	April 2006/ December 2012	Six years	Infection in NICU	Healthcare-associated sepsis is defined as a positive blood culture taken after 48 h of admission to the NICU with the presence of clinical signs that are suggestive of neonatal septicemia. Each neonate showing sepsis was subjected to sampling for blood culture, cerebrospinal fluid (CSF) culture, or other samples according to the clinical presentation.	22	*C. albicans*	“The rate of healthcare-associated infections in the neonatal intensive care unit at KAASH was relatively high. In addition, the mortality rate was observed to be high (27.1%) due to the causative organisms’ high virulence”.
Afify 2012 [43]	Case–Control study	-	-	33	18	-	Vaginal: 19, C/S: 14	2010/ 2011	Two years	Ventilator-associated pneumonia	-	one	*C. albicans*	“In conclusion, risk factors for the development of VAP include (1) decreased body weight and gestational age, (2) increased duration of NICU admission, MV, and use of invasive maneuvers, (3) hypothermia, mucopurulent ETT secretions and the use of inotrops/corticosteroids, (4) raised serum CRP, hypoalbuminemia and positive blood cultures and (5) nosocomial infection by *Klebsiella*, *Pseudomonas*, *Staph aureus*, *E. coli*, and Candida”.

Abbreviations: CS = cesarean section, NS = not specified, NICU = Neonatal Intensive Care Unit.

## Data Availability

Data are available from the corresponding author upon reasonable request.

## Supplementary Materials

The following supporting information can be downloaded at: https://www.mdpi.com/article/10.3390/diseases12070154/s1, Table S1: The Cochrane Collaboration’s tool for assessing risk of bias. Table S2: NIH Quality Assessment Tool for Observational Cohort and Cross-Sectional Studies. Table S3: Assessment Tool for Case Report Studies.
