# Supplementary material for: Prevalence and Species Distribution of Neonatal Candidiasis: A Systematic Review and Meta-Analysis
Source: Diseases. 2024 Jul 12;12(7):154. doi: 10.3390/diseases12070154 (PMC11276108; doi:10.3390/diseases12070154)
Supplement: Supplementary file 1 [file diseases-12-00154-s001.zip › diseases-3063687-supplementary.pdf]

Supplementary Materials

Table S1. The Cochrane Collaboration's tool for assessing risk of bias.

Study No	Random Sequence Generation (Selection Bias)	Allocation Concealment (Selection Bias)	Blinding of Participants and Personnel (Performance Bias)	Blinding of Outcome Assessment (Detection Bias)	Incomplete Outcome Data (Attrition Bias)	Selective Reporting (Reporting Bias)	Other Bias	Overall
	**Low\High\Unclear risk of bias**	Low\High\Unclear risk of bias	**Low\High\Unclear risk of bias**	Low\High\Unclear risk of bias	**Low\High\Unclear risk of bias**	Low\High\Unclear risk of bias	**Low\High\Unclear risk of bias**	
**Mersal 2013**	Low	Unclear	Unclear	Unclear	Unclear	Unclear	Unclear	Unclear
**Mohamed 2012**	Low	Low	Low	Unclear	Unclear	Unclear	Unclear	Unclear

Table S2. NIH Quality Assessment Tool for Observational Cohort and Cross-Sectional Studies.

ID	1. Was the research question or objective in this paper clearly stated?	2. Was the study population specified and defined?	3. Was the participation rate of eligible persons at least 50%?	4. Were all the subjects selected or recruited from the same or similar populations (including the same period)? Were inclusion and exclusion criteria for being in the study prespecified and applied	5. Was a sample size justification, power description, or variance and effect estimates provided?	6. For the analyses in this paper, were the exposure(s) of interest measured before the outcome(s) being measured?	7. Was the time frame sufficient so that one could reasonably expect to see an association between exposure and outcome if it existed?	8. For exposures that can vary in amount or level, did the study examine different levels of the exposure as related to the outcome (e.g., categories of exposure or	9. Were the exposure measures (independent variables) clearly defined, valid, reliable, and implemented consistently across all study participants?	10. Was the exposure(s) assessed more than once over time?	11. Were the outcome measures (dependent variables) clearly defined, valid, reliable, and implemented consistently across all study participants?	12. Were the people assessing the outcomes blinded to the participants' exposures/interventions?	13. Was the loss to follow-up after baseline 20% or less?	14. Were key potential confounding variables measured and adjusted statistically for their impact on the relationship between exposure(s) and outcome(s)?	Total scores

				**uniformly to all participants?**				**exposure measured as a continuous variable)?**							
	**Yes/No/Not reported (NR) or cannot determine (CD) or not applicable (NA)**	**Yes/No/Not reported (NR) or cannot determine (CD) or not applicable (NA)**	**Yes/No/Not reported (NR) or cannot determine (CD) or not applicable (NA)**	**Yes/No/Not reported (NR) or cannot determine (CD) or not applicable (NA)**	**Yes/No/Not reported (NR) or cannot determine (CD) or not applicable (NA)**	**Yes/No/Not reported (NR) or cannot determine (CD) or not applicable (NA)**	**Yes/No/Not reported (NR) or cannot determine (CD) or not applicable (NA)**	**Yes/No/Not reported (NR) or cannot determine (CD) or not applicable (NA)**	**Yes/No/Not reported (NR) or cannot determine (CD) or not applicable (NA)**	**Yes/No/Not reported (NR) or cannot determine (CD) or not applicable (NA)**	**Yes/No/Not reported (NR) or cannot determine (CD) or not applicable (NA)**	**Yes/No/Not reported (NR) or cannot determine (CD) or not applicable (NA)**	**Yes/No/Not reported (NR) or cannot determine (CD) or not applicable (NA)**	**Yes/No/Not reported (NR) or cannot determine (CD) or not applicable (NA)**	
**Alfaleh 2010**	Yes	Yes	No	Yes	No	No	Yes	NA	Yes	NA	Yes	NR	Yes	NA	**8.5**
**Alharbi 2022**	yes	Yes	Yes	Yes	No	No	Yes	NA	Yes	NA	Yes	NR	Yes	NA	**9**
**Aljasser 2004**	Yes	Yes	Yes	Yes	No	No	Yes	NA	Yes	NA	Yes	NR	Yes	NA	**9**
**Almoosa 2017**	Yes	Yes	Yes	Yes	No	No	Yes	NA	Yes	NA	Yes	NR	Yes	NA	**9**
**Almouqdad 2019**	Yes	Yes	Yes	Yes	Yes	Yes	Yes	NA	No	NA	Yes	NR	Yes	Yes	**10.5**
**Al-Matary 2019**	Yes	Yes	Yes	Yes	No	No	Yes	NA	Yes	NA	Yes	NR	Yes	NA	**9**
**Al-Matary 2022**	Yes	Yes	Yes	Yes	No	Yes	Yes	NA	CD	NA	Yes	NR	Yes	NR	**8.5**
**Ohlsson 1997**	Yes	Yes	Yes	Yes	No	Yes	Yes	NA	Yes	NA	Yes	NR	Yes	No	**10**
**Eisi 2022**	Yes	Yes	Yes	Yes	Yes	Yes	Yes	NA	Yes	NA	Yes	yes	Yes	Yes	**12**
**Elbashier 1998**	Yes	Yes	Yes	Yes	No	Yes	Yes	NA	Yes	NA	Yes	NR	Yes	NA	**9.5**
**Elbashier 1994**	Yes	Yes	Yes	Yes	No	Yes	Yes	NA	Yes	NA	Yes	NR	Yes	NA	**9.5**
**Afifi 2012**	Yes	Yes	Yes	Yes	No	No	Yes	NA	Yes	NA	Yes	NR	Yes	Yes	**10**
**Alhussaini 2016**	Yes	Yes	Yes	Yes	No	Yes	Yes	NA	Yes	NA	Yes	NR	Yes	Yes	**10.5**
**Al-Zahrani 2013**	Yes	Yes	Yes	Yes	No	Yes	Yes	NA	Yes	NA	Yes	NR	Yes	No	**10**
**Faraz 2019**	Yes	Yes	Yes	Yes	No	Yes	Yes	NA	Yes	NA	Yes	NR	Yes	No	**10**
**Ndlovu 2021**	Yes	Yes	Yes	Yes	No	No	Yes	NA	Yes	NA	Yes	NR	Yes	NA	**9**

Table S3. Murad et al. 2018 Assessment Tool for Case Report Studies.

**ID**	**1. Does the patient represent the whole experience of the investigator (center), or is the selection method unclear to the extent**	**2. Was the exposure adequately ascertained?**	**3. Was the outcome adequately ascertained?**	**4. Were other alternative causes that may explain the observation ruled out?**	**5. Was there a challenge/rechallenge phenomenon?**	**6. Was there a dose-response effect?**	**7. Was follow-up long enough for outcomes to occur?**	**8. Is the case described with sufficient details to allow other investigators to replicate the research or to allow**	**Total scores: Yes = 1 // No = 0.5 // NR & NA & CD = 0**	**Quality rating: good (6.5-8points), fair (5-6.5points), or poor (4.5-0 points)**

	that other patients with similar presentation may not have been reported?							practitioners to make inferences about their practice?		
	**Yes/No/Not reported (NR) or cannot determine (CD) or not applicable (NA)**	**Yes/No/Not reported (NR) or cannot determine (CD) or not applicable (NA)**	**Yes/No/Not reported (NR) or cannot determine (CD) or not applicable (NA)**	**Yes/No/Not reported (NR) or cannot determine (CD) or not applicable (NA)**	**Yes/No/Not reported (NR) or cannot determine (CD) or not applicable (NA)**	**Yes/No/Not reported (NR) or cannot determine (CD) or not applicable (NA)**	**Yes/No/Not reported (NR) or cannot determine (CD) or not applicable (NA)**	**Yes/No/Not reported (NR) or cannot determine (CD) or not applicable (NA)**		
Abuhajj 2021	NR	No	Yes	Yes	NA	NA	NA	Yes	3.5	Poor
Al Arishi 1997	NR	CD	Yes	CD	No	Yes	Yes	Yes	4.5	Poor
Azhar 2012	NR	Yes	Yes	Yes	NR	NR	Yes	Yes	5	Fair
